# Food insecurity amongst asylum seekers and people without status in Israel

**DOI:** 10.1186/s13584-024-00622-y

**Published:** 2024-08-12

**Authors:** Moran Blaychfeld-Magnazi, Zohar Mor, Gaya Sartena, Rebecca Anne Goldsmith, Einat Ophir, Ronit Endevelt

**Affiliations:** 1 Ministry of Health, Public Health Services, Jerusalem, Israel; 2 Department of Health, Tel Aviv, Ministry of Health, Tel Aviv, Israel; 3 School of Health Sciences, Ashkelon Academic College, Ashkelon, Israel; 4 Faculty of Welfare and Health, School of Public Health, University of Haifa, Haifa, Israel

**Keywords:** Food insecurity, Asylum seekers, COVID-19, Migrants, Israel

## Abstract

**Background:**

The COVID-19 pandemic caused massive disruptions globally, with food insecurity a primary concern amongst vulnerable communities. As one of the most marginalized and vulnerable groups in Israeli society asylum seekers and undocumented populations were amongst the first to be affected by the pandemic and the economic crisis that followed. The objective of the study was to evaluate the severity and causes of food insecurity among asylum seekers and other undocumented communities because of COVID-19.

**Methods:**

A multi method approach was used. The quantitative component included an online questionnaire regarding access to food, aid and choices, and the 6 item Household Food Security Survey Module (HFSSM) The qualitative component included 4 focus groups and thematic analysis. The study was conducted in November 2020, by the Ministry of Health's Nutrition Division and the Tel Aviv Municipality's foreign community assistance and information center (Mesila). The convenience sample was drawn from the low-income neighborhood population of South Tel Aviv. Logistic regression, multivariate analysis and content analysis, were performed.

**Results:**

Four hundred eighty-five people completed the quantitative survey, with average age 33.2 ± 5.4 years and 349 (72.0%) experienced food insecurity. In the multivariate analysis, being older (*p* = 0.04, Odds Ratio OR 1.1, Confidence Interval CI 1.05–1.15) and being single (unmarried) (*p* = 0.03, OR 2.1, CI 1.2, 3.5) predicted food insecurity. Qualitative findings identified three main themes: children preferring Israeli/ Western foods to traditional foods; financial stresses were compounded; a preference for receiving assistance with purchasing food (vouchers), rather than food handouts.

**Conclusion:**

In conclusion, vulnerable populations (asylum seekers and other undocumented communities) were severely affected and are in danger of food insecurity. Culturally relevant and contextualized solutions are needed to address the acute hunger within the community. These include establishment of a cross-ministerial forum, a social grocery store, increased liaison with food rescue bodies, complete nutritional support for children in educational settings and increased guidance regarding food choices and budgeting.

**Supplementary Information:**

The online version contains supplementary material available at 10.1186/s13584-024-00622-y.

## Introduction

### Background

The SARS-CoV-2, the virus causing COVID-19, has caused massive disruptions globally in people's lives. The impact of the virus has disproportionately affected marginalized groups and those in fragile and under-resourced settings [1]. Such populations have been deeply affected by the pandemic, with food insecurity being a primary concern (https://www.usglc.org/coronavirus/globalhunger/#:~:text=Today%2C%20the%20number%20of%20severely,rise%20to%20323%20million%20people) [2]. The ability to feed oneself and one's family adequately is a human right linked to the right to life and dignity, however in times of crisis this right is often compromised [3]. People seeking asylum are vulnerable to food insecurity due to limited opportunities for social and economic participation [4], and the disruption of food security amongst asylum seekers and people without status living in Israel has been evident from the onset of the pandemic. This was most likely due to significant reduction in employment opportunities and resultant loss of employment. As such, this prompted the Ministry of Health's Nutrition Division and the Tel Aviv-Yafo Municipal Welfare Unit for the foreign community, via the NGO, Mesila, to conduct a study to analyze the severity of food insecurity within these communities and explore viable solutions. Mesila, (a Hebrew acronym) is an NGO, which provides assistance and social services of many types (education, health etc.) to asylum seekers and undocumented persons in the greater Tel Aviv area. According to the United States Department of Agriculture (USDA), food insecurity is a condition in which households lack access to healthy and adequate food because of limited money or other resources. The USDA quantifies food insecurity based on responses to a validated series of eighteen survey questions and statements, ('The Household Food Security Survey Module- HFSSM) or the validated shortened version, with six questions [5–7]. Based on survey responses, the USDA ranks households into four categories: 1) *High food security*, in which all household members have uninterrupted access to enough food to allow for an active and healthy life(score = 0); 2) *Marginal food security*, in which some of the household members reported anxiety about insufficient food or shortage of food in the household, but there was no indication of changes in diet or food intake (score = 1); 3) *Low food security*, in which there is reduced quality or variety of food in the household, but not necessarily resulting in reduced food (scores 2,3,4); 4) *Very low food security*, this includes multiple indications of disrupted eating patterns and reduced food intake(scores 5,6) [8].

An alternate classification, according to HFSSM documentation, is by combining high and marginal scores (0,1) and labelling this as “food secure” whereas scores of 2–6 are “food insecure”, with 2 levels—low food security, scores 2–4, and very low food security, scores of 5–6.". This alternate classification was used.

Families living with food insecurity can also cycle between periods of food insufficiency and periods of relative stability or even abundance. Related studies demonstrate links between food insecurity and increased stress, leading to harmful diet- related behaviors [6]. Coping with food insecurity often includes reliance on low cost, mainly ultra-processed foods, and tendencies to overeat or binge when resources are available. Furthermore, qualitative studies demonstrate that children are acutely aware of their household’s food insecurity, resulting in psychological ramifications.

### Asylum seekers and people without status in Israel

According to the United Nations High Commissioner for Refugees (UNHCR), as of 2021 there were approximately 41,000 asylum seekers, mainly from Eritrea and Sudan, and people without status living in Israel, around 14,000 of which live in the Tel Aviv area [9, 10]. In accordance with the principle of non-refoulement, Eritrean and Sudanese are permitted to stay in the country temporarily under a non-removal policy, or group protection.^1^ Since the beginning of the COVID-19 pandemic in Israel, the plight of asylum seekers and people without status has been amplified; these communities continue to experience vastly disproportionate health, economic and social impacts. Due to the increase in COVID-19 cases, the Israeli government executed lockdowns and enforced movement restrictions. These lockdowns included the closure of all non-essential businesses, including restaurants, hotels, and all in-person customer-based businesses. Even as restrictions slowly lifted, the hotel and restaurant industries remained at a halt; many asylum seekers typically find employment in these industries, in kitchens, and cleaning hotel rooms across the city of Tel Aviv.

Asylum seekers and undocumented persons are not entitled to state social security, including health insurance or welfare benefits. Labor migrants with valid work visas are entitled to health insurance. In general, many of their needs, including nutritional needs. are not adequately met by government instrumentalities. Therefore, lockdowns and associated loss of livelihoods meant thousands of families and individuals did not have the financial means to access food and other necessities. During this emergency period asylum seekers were reliant on aid from Non-Governmental Organizations (NGOs), and private citizens. Particularly in the beginning of the pandemic, there was limited organized assistance given by government instrumentalities or the National Insurance Institute to assist these individuals, who found themselves, often overnight, without employment and with no available sources of money for food, rent etc. To address the growing need for food support amongst the community, Mesila began distributing dry food packages and supermarket vouchers.^2^ Despite these increased efforts, gaps remained in mitigating the acute hunger the community was vocalizing. As the pandemic persisted, a partnership was established in June 2020 between the Tel Aviv Department of Health, the research department of the Ministry of Health's Nutrition Division and the Tel Aviv Municipality's Foreign Community Assistance and Information Center. The goal was assessing the level of food insecurity within the asylum seeker community and determining how best to develop culturally relevant and sustainable solutions.

There is a dearth of material published regarding food insecurity in these communities. In an attempt to address this important issue, this paper presents the results of a study conducted between September 2020—December 2020, among asylum seekers and people without status in Tel Aviv during the COVID-19 pandemic.

## Methods

In order to gain a comprehensive understanding of the level of food insecurity among migrants from different countries in Tel Aviv during the COVID-19 pandemic, a multi-method approach was used. A quantitative analytical cross-sectional study was conducted amongst a convenience sample of 485 asylum seekers and other undocumented people who live in Tel Aviv and use the internet. In addition, the study included a qualitative research component and integrated data collected from four focus groups from various targeted sub-groups within the population.

The study was multi-method, and the quantitative and qualitative methodology are described separately.

### Quantitative methodology

#### Sampling procedures

A convenience sample was drawn from those whom Mesila reaches by way of social media, through WhatsApp groups managed by community leaders and NGOs working with the target population. This method was chosen based on Mesila*'s* experiences, and its use of social media platforms, including Facebook, having found these to be effective methods of communicating with these populations. Other NGO's, including ASSAF, the organization for assistance to refugees and asylum seekers in Israel, were approached. In addition, the asylum seekers and undocumented persons have their own Whatsapp group and this was also used. Though the exact number of those accessing these social media platforms and WhatsApp groups is unknown, it is known that these are the preferred methods of communication, and are widely used.

The following text was posted on the WhatsApp groups and the Facebook groups:

- "The corona plague has caused many to find themselves without a job and a livelihood. During this period, many are facing nutritional challenges. This questionnaire is part of a study, in which we seek to assess the number of people in the community of refugees and migrant workers who need assistance and what type of assistance is required. Filling out the questionnaire will help us explore ways to help the community in general.
- At the end of the questionnaire, we will also offer you to fill in personal details in case it is possible to get assistance from the local authority.
- The researchers will not have access to the identifying data and the findings will be published without identifying details.
- Thank you for filling out the short questionnaire".

#### Study population

The study population included all asylum seekers and undocumented adult individuals (aged 18 or older) who elected to complete the survey online and who provided informed consent.

#### The survey

In November 2020, an online survey based on the USDA instrument termed “ The Household Food Security Survey Module (HFSSM)” [7], was distributed to the target populations through Mesila*'s* community network and social media channels. The survey included 18 general questions and 6 validated questions of the HFSSM regarding the state of a respondent's food insecurity for the six-month period (from May 2020) preceding the survey. The HFSSM was chosen as the instrument for assessing food insecurity, as it has been validated for several populations, and is used in many countries, thus facilitating comparisons.

The survey was translated from the original English text to Tigrinya and Arabic by native Tigrinya or Arabic speakers fluent in English.

The survey included questions in which respondents were instructed to indicate how often the statements were true, for example: “The food that (I/we) bought just didn’t last and (I/we) didn’t have money to get more”; (I/we) couldn’t afford to eat balanced meals, and in the last 12 months, were you ever hungry but didn’t eat because you couldn’t afford enough food?” Demographic questions and questions about the type of food assistance received were also included. The questionnaire is included in the Supplementary Materials.

#### Inclusion criteria

All respondents who completed the online survey in any of the survey languages (English, Tigrinya, and Arabic) were included in the results.

#### Exclusion criteria

Subjects who indicated that their age was younger than 18 years of age, those without access to internet, and those who did not provide informed consent were excluded from the results.

#### Statistical analyses

The Food Insecurity Scale in this cross-sectional study was aggregated as follows: marginal food security (levels 0–1), low food security (level 2–4), or very low food security (level 5–6) [11].

Categorical variables were compared by the Chi-square test while continuous variables were compared by the Student's t-test where the distribution was normal and by the Mann–Whitney test for non-normal distributions. Variables with *p* values less than 0.05 in the univariate analyses were included in the multivariate analysis to identify variables predicting food insecurity by logistical regression outlined by odds ratios (OR) and 95% confidence intervals (95% CI).

### Qualitative methodology

In addition to the survey, four focus groups were conducted. Participants were recruited through Mesila*’s* network of clients and community activists. The four groups were: 1) community activists and influencers (men and women); 2) Eritrean women; 3) Sudanese women; 4) West Africa and Philippines (mixed men and women). Each group had 6–12 participants and was conducted in Hebrew and English with translations available in Arabic and Tigrinya. Each focus group lasted about 2 h and was facilitated by both Mesila staff and Nutrition Division staff. The focus group discussions covered topics related to daily eating habits, dietary needs, and reflections and feedback regarding food assistance they received from NGOs and other groups. Some of the discussion questions included: Did they experience food insecurity and nutrition challenges during “normal” times, and how had this changed in recent months; did they receive direct food assistance (DFA), and if so, from whom; their opinion about the DFA received; which foods they usually receive; what are their typical food consumption habits; how much food is required per day; their preferred type of assistance; is additional external assistance needed; what people are doing in order to cope with the situation; who in the community is in greatest need of assistance.

#### Data analysis

The content analysis approach was used for data analysis [12]. This process involved several distinct stages, beginning with familiarization with the data. The narratives from the different participant groups were analyzed separately to determine the key themes. The analysis involved indexing and sifting through the data and sorting quotes from participants; charting or selecting quotes and placing them in the appropriate thematic category; comparing the findings in all four focus groups; and developing a final interpretation.

The study was approved by the Institutional Ethics Board of the University of Haifa, Israel, 24/9/2020, No. 436/21.

## Results

Four hundred eighty-five respondents completed the questionnaire (406 in Tigrinya, 68 in English and 11 in Arabic), their average age was 33.2 ± 5.4 years. The majority (74.5%) were women, and 87.2% reported that they have children in Israel (an average of 2.6 ± 1.2 children) (Table 1).

**Table 1:** Demographic characteristics of respondents

Criteria		N (Percent %)	Mean ± standard deviation
Age			33.16 ± 5.45
Number of years in Israel			10.07 ± 3.9
Country of origin	Eritrea	410 (84.4)	
	Sudan	14 (2.9)	
	Philippines	15 (3.1)	
	India	5 (1)	
	Former USSR	2 (0.4)	
	Ethiopia	21 (4.3)	
	Other	17 (3.5)	
	Missing	2 (0.4)	
Gender	Male	124 (25.5)	
	Female	362 (74.5)	
Marital Status	Single	55 (11.3)	
	Married	330 (67.9)	
	Divorced	65 (13.4)	
	Widowed	8 (1.6)	
	In an informal relationship	21 (4.3)	
	Separated because of the circumstances	7 (1.4)	

Most of the study participants originated in Eritrea, and were more commonly younger, married and spent shorter time in Israel as compared with those who came from other countries. Eritrean were more likely to use food vouchers.

As shown in Table 2, 418 reported food insecurity (262 severe food insecurity, 156 moderate food insecurity), while 67 had enough food (USDA categories 5–6, 2–4 and 0–1, respectively). Those who experienced food insecurity were more commonly single (unmarried) and non-Eritreans (Table 3). Those who had families (living with spouse and/or children and/or were receiving food aid from organizations were more likely to be food secure.

**Table 2:**
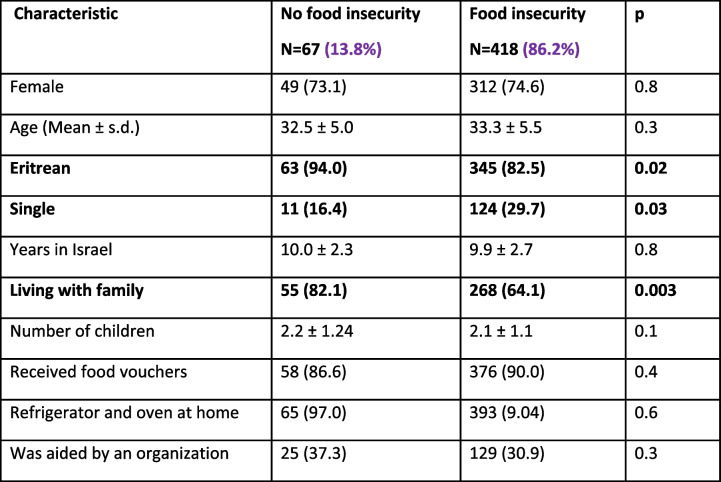
Comparison between respondents in Israel who experienced food insecurity during the COVID outbreak with those who have not experienced food insecurity

(Results significant at *p* <  = 0.05 are highlighted)

**Table 3:** Comparison between study participants who originate in Eritrea to those who came from other countries

Characteristic	Eritrea 480 (84.4%)	Other countries 75 (15.6%)	p
Female	301 (73.8)	60 (77.9)	0.2
Age (Mean ± s.d.)	32.5 ± 4.2	36.5 ± 8.8	< 0.001
Single	91 (22.3)	44 (57.1)	< 0.001
Years in Israel	9.8 ± 1.4	11.0 ± 5.7	< 0.001
Living with family	292 (71.6)	31 (40.3)	< 0.001
Number of children	2.4 ± 0.7	1.5 ± 1.2	0.2
Received food vouchers	381 (93.4)	53 (68.8)	< 0.001
Refrigerator or oven at home	382 (93.6)	76 (98.7)	0.1
Was aided by an organization	133 (32.6)	21 (27.3)	0.4

When asked about the type of food assistance received, 44% of the respondents noted that they received assistance from a local NGO. The main type of assistance came in the form of supermarket vouchers (74.5%). In some of the cases, the respondents received prepackaged boxes of canned and dry food. (9.3%). When asked about the type of assistance they prefer to receive, most (90%) reported that they preferred to receive supermarket vouchers rather than prepackaged DFA.

In the multivariate analysis, being older (*p* = 0.04, Odds Ratio OR 1.1, CI 1.05–1.15) and being single (unmarried) (*p* = 0.03, OR 2.1, CI 1.2, 3.5) predicted food insecurity (Table 4).

**Table 4:** Multivariate analysis of characteristics predicting food security

	**OR (95% CI)**	**p**
Age	1.1 (1.05–1.15)	0.04
Eritrea	0.6 (0.3–1.4)	0.2
Time in Israel	0.9 (0.8–1.0)	0.08
Single	2.1 (1.2–3.5)	0.03
Receiving food basket	0.9 (0.5–1.6)	0.7

### Qualitative findings

Qualitative findings are based on the four focus groups. The groups were comprised as follows: Group 1: eight community leaders from Eritrea, Sudan, Philippines, Ivory Coast; Group 2: six clients of Mesila from Eritrea; Group 3: 12 Sudanese, clients of Mesila and referrals from community leaders of additional people in need; Group 4: six people from the Ivory Coast and the Philippines, who have not necessarily approached Mesila for regular assistance prior to the pandemic.

The demographic makeup of the focus groups is shown below, in Table 5

**Table 5:** Demographic characteristics of focus group participants

Variable	*N* = 32
Gender (Male, Female)	8,24
Average age (Years)	37.9 ± 9
Years of Education	11.7 ± 7 (range from 0–17)
Employment in Israel	Cleaning—13
	Caring for the elderly—1
	Assistant in kindergarten—1
	Translation—4
	Other—7
	Unemployed—6

Results were categorized into four themes, as follows (Table 6):

- 1. **Eating habits in general** – Before the pandemic, most ate at home, adapting mealtimes to their working hours, mainly in the evening, when they returned from work. The adults adhere to eating traditional dishes, which differ in each country of origin, whereas the children prefer "Israeli/Western food."
  Eritrea: the basic meal is *injera*, made from *teff* flour, which is a fermented flatbread with a slightly spongy texture and eaten with various sauces prepared with legumes, vegetables or meat.
  Sudan: meat is a central part of the menu.
  Philippines: rice is the basis for every meal, and various soups, eggs, fish, bok choy and tofu are included.
- 2. **Eating habits during the COVID-19 pandemic** – it has been quite difficult to acquire food and hard to meet the demands of the children for "Israeli food". Obtaining products, such as *teff flour* and certain spices, from the countries of origin was difficult*.* In addition, participants reported difficulties, due to high prices, in accessing food that their children preferred. Breakfast is usually limited, and the adults normally do not eat lunch. They expressed shame and self-blame for not being able to provide their children the food items they liked.
- 3. **The type and relevance of food assistance received** — the prepackaged DFA distributed is appreciated but does not necessarily suit the population. The community received cooked food from donations, but it often did not meet their habits and preferences. They also received vouchers to buy food in supermarkets, as well as other essential items, such as laundry detergent.
- 4. **The community's preferences should be taken into consideration** when deciding on the type of what kind of assistance provided —they suggested several ways to maximize the support:
  - Distribution of dry food packages and vegetables, fruit, and dairy products. It was emphasized that the community should be consulted on the products and quantities included. Products provided must be adapted to people with different health conditions, such as diabetes. Furthermore, the focus group of community leaders and activists stated that a dry food package is better than a supermarket voucher, primarily due to concerns that some people may use the vouchers to buy non-essential items, such as cigarettes and alcoholic beverages. However, some supported the idea of vouchers as this enabled independence in purchasing decisions.
  - Distribution of supermarket vouchers enabled people to purchase basic essential items such as diapers and infant formula. The vouchers should contain guidelines and restrict the purchase of non-essential or harmful items, such as cigarettes and alcohol.
  - Provision of sandwiches for children in kindergartens and schools- some participants mentioned children come to kindergarten and school without eating anything at home and did not eat a meal all day.

**Table 6:** Summary of key focus group themes and selected comments

	Major Themes	Sub-Themes	Testimonials
1	**Eating habits in general – the adults eat traditional dishes from their countries of origin, whereas the children prefer "Israeli/Western food"**	a. At home, most of the food is traditional cooking b. The adults eat basic food, and sometimes meat. Over time there is a combination of local "Israeli” food in the menu c. The children were exposed to Western food in Israel, mostly processed. They prefer not to eat traditional food	*“The children do not always eat what we are used to in Sudan. They were born here; they want cheese, eggs, schnitzels, cornflakes, chicken breast”*
2	**During the COVID-19 pandemic – it has been quite difficult to acquire food and hard to meet the demands of the children for "Israeli food"**	a. Economic difficulty- to obtain “Israeli food”: the food that the children prefer b. Difficult to obtain traditional food, which did not arrive countries of origin due to supply chain disruptions c. On the one hand a desire to please the children and buy them the food they want, and on the other hand a difficulty in setting boundaries for the children around their food behavior d. Difficulty in accessing food due to financial constraints is an additional challenge to deal with, in addition to the usual challenges of rent and health insurance payments, which worries them	*“Things do not come from Africa because of the COVID-19 pandemic; we miss a lot of things.”* *Mother “I have a girl at the age of 3, she asks for more than her older sisters and brothers. She wants milk in the morning, schnitzel* *Child replies: now now now* *Mother: No money, now bring urgent, my heart really hurts. If I don’t have one, I'll bring it. This small bag of schnitzel it’s two days and it’s over. I bring what I have. If there isn’t there isn’t. I now go to the supermarket I buy like this. I continue living, I tell her I don’t have* *Child replies:, you have money, bring me”* *“Parents avoid talking about the difficulty because they are afraid of gossip”*
3	**The food assistance that has been extended while dealing with the COVID-19 is appreciated, but does not necessarily suit the population**	a. Gratitude- Any assistance received is appreciated, however, inconsistency between the food they received and the taste and habits of the community	*“We have seen people who cannot pay rent, so without money for a place to live, you can’t spend money for food”*
4	**The community's preferences should be taken into consideration when deciding what kind of assistance is given**	a. There is a preference for grocery store vouchers that can be used at large supermarkets so they can freely choose what to buy b. If food boxes are provided – it is important to consult with the community in advance c. Cooked food is not appropriate due to differences in tastes and preferences d. They would like sandwiches and fruits to be given to the children who attend educational institutions	*“Now that you’re starting with a new program it’s good to know what’s good for us”* *“If you give me the voucher I will buy what the children want, if we get the food itself, the children will just eat it”* *“In Africa they give food in the morning at school, why not here? A sandwich and fruit can be excellent”*

## Discussion

The research findings indicate varying levels of food insecurity among the respondents, with significant severe food insecurity, thus indicating a need to urgently and comprehensively address this situation. Previous research on food insecurity in Israel does not include the asylum seeker community or people without status. The research objective and the correlating findings focus on highlighting the severity of food insecurity within the asylum seeker community in Tel Aviv during a very specific time as a result of the secondary socio-economic impacts of the COVID-19 pandemic. There is no available data about this community regarding food insecurity before the pandemic. However the sudden and prolonged loss of employment and income resulted in a worsening of food insecurity, as described by the subpopulations and as observed by Mesila, indicated by increased number of calls for assistance. A cross-sectional study of this nature cannot establish cause and effect or report trends, though, given the social-economic aspect, the situation is most likely chronic, and was exacerbated during the pandemic.

A more robust study would need to be conducted to delve deeper into the causation of food insecurity and gaps in nutrition within the community—such as; a vulnerability analysis to understand protections risks and negative coping mechanism; a gender analysis both in terms of women’s needs and women’s role in the consumption of a healthy diet; dietary and behavioral analysis to understand significant gaps in nutrition and how behaviors and cultural norms may influence results; a food consumption score nutritional analysis assessing the nutritional value of the households diet, and a household dietary diversity analysis reflecting a households economic ability to consume a variety of foods (https://resources.vam.wfp.org/data-analysis/quantitative/food-security/food-consumption-score) [13]. Additionally an analysis of current government policies which hinder the socioeconomic development and social protection of asylum seekers and undocumented people in Israel is needed, so as to determine a nuanced and sustainable solution to food insecurity amongst the population.

This study attempted to examine, in real time during the Covid 19 pandemic, the level of food insecurity of asylum seekers and people without status living in Tel Aviv; the target population already living in a stressed socio-economic state pre- pandemic. Though many of those in these communities worked prior to the pandemic, their work was generally in the service sector, and was not well paid, and they lacked job security. The study examined the severity of food insecurity, and suggestions from community members on ways to alleviate their situations.

There is little data on this specific group, though studies have related to food security changes during the pandemic period The study by Maisel (2021) [14] and the report by Latet [15] (a food assistance NGO) covering the same period in 2021 both indicated worsening levels of food security as a result of the Covid 19 pandemic, due to increased unemployment. In addition, school age children who had previously received food assistance (via school lunch programs etc.) no longer did, as schools were closed for significant periods of time. The reports also cited information from NGO’s, such as Mesila, regarding increased requests for assistance, including from families who had not, prior to the pandemic, requested assistance. The abilities of the NGO’s to respond were hampered by the lockdowns, reduced manpower in the agricultural sector and interruptions in food supply chains. Latet reported that in 2016 a national food security survey showed 18% food insecurity, whereas, using the same tools, in 2021 the level jumped to 25%, with the severe level going to 12% from 9%.

The literature on the topic of asylum seekers and food security report that asylum seekers are generally more vulnerable to food insecurity, even prior to the pandemic. In a study conducted in Melbourne, Australia in 2015, among 56 asylum seekers, the researchers found that most asylum seekers had some form of food insecurity, while almost 40% experienced food insecurity with hunger. Furthermore, they experienced food insecurity regardless the length of time in Australia. About 50% stated that the food they received was sufficient, and acceptable, whereas one third stated that that they would eat the food, but it was not the food that they preferred [16].

Another study in Norway 2019, which included 205 asylum seekers, reported that 93% suffered from food insecurity, of whom 78% reported food insecurity. In this study, the researchers found a higher odd of experiencing food insecurity in men as compared to women [17].

A Turkish report from 2020 "On forced migrants and the COVID-19 pandemic” stated that before the COVID-19 pandemic asylum seekers in the country were given the same rights in accessing healthcare as citizens on the condition that they must be registered with the Directorate General of Migration Management (DGMM) [18]. However, although they had the right to access healthcare, they experience different barriers in access to services because of legal status, language barriers, lack of knowledge about how the health system functions, economic barriers and healthcare workers’ negative attitudes towards them. In Israel, the asylum seekers received assistance from volunteer organizations and from the government.

In our study, we found that the levels of severe food insecurity were more common among those who were single. These findings are similar to other studies that show one of the main characteristics of food insecurity are being single or divorced [19, 20]. In our study, those living in a family setting and/or receiving some form of aid, (food or other), were less likely to be food insecure (*p* < 0.01). This connection was found in a few studies, though in the literature there are also contradictory reports, which state that, those who lived with children were more likely to be newly at risk for food insecurity [21–24].

There are various ways to alleviate or mitigate food insecurity, such as food vouchers, food baskets, food trucks, school feeding programs that deliver supplemental food to children in the form of meals etc [4, 25]. In our study, the questionnaire asked the participants as to the type of assistance they preferred to receive. Most participants (90%) said they prefer supermarket vouchers to prepackaged DFA (Direct Food Assistance). A study conducted in Kenya in 2012, analyzed which aid the participants preferred receiving, whether money, fresh food or vouchers. They found that 32% preferred cash, 26% preferred fresh food, 22% preferred a mix of transfer types, and 20% preferred vouchers. Among those who preferred a mix of the three primary forms, 40% indicated all three, namely cash, food and vouchers [26].

An Ecuadorean study from 2014 examined which form of assistance is better: Cash, food or vouchers.

All three forms of assistance significantly improve a household’s food consumption and non –food consumption. When a household receives food and food vouchers, there is a significant increase in the outlay for non-food consumption [27].

In the second part of our study, four focus groups were convened. The community leaders group mentioned a concern that if people receive food vouchers, they will purchase items other than necessary food. Therefore, they recommend receiving a dry food package. In the other focus groups, most of the participants indicated that they were interested in receiving a cash slip, to enable autonomy in deciding on purchasing. Purchasing power and autonomy over one's food choices and diet are extremely important elements to consider, as these promote independence, self- efficacy, and self- reliance, and support the dignity of the community, rather than outside sources dictating one's diet and nutrition through pre-packed food aid [28–30].

The adult asylum seekers ate traditional foods, while their children were accustomed to the Israeli foods, which sometimes were more processed and expensive than the traditional foods. During the first lockdown in Israel, most of the asylum seekers lost their livelihoods overnight with no financial compensation or subsistence provided by the government. Asylum seekers were dependent on assistance from NGOs, though it was not enough money to buy the foods their children wished to eat. Many were very appreciative of the assistance received, even if some assistance did not match their tastes. A few stated that they preferred to remain hungry and discard (throw out) the food rather than eat the food to which they were not accustomed.

The recommendations outlined are based on the findings of this study. However, the method and practicality of implementation of the recommendations needs to be assessed carefully. When implementation does occur, monitoring and evaluation are needed and undoubtedly, a repeat study, albeit limited, would provide important information, including as to how serious the problem still is, if at all.

## Study limitations

Some limitations exist regarding the research findings, given the emergency nature of the situation and the urgent need to conduct the study with limited resources, and time constraints. There is a potential sampling bias because the survey was distributed as a link on social media and through WhatsApp channels, and internet access was needed in order to complete the survey. Therefore, the study population included people with an internet connection and those who are literate, use social media, and can complete a digital survey. One could surmise that if there was food insecurity in this group, then most likely in those without internet access and/or less literate, there may be even greater incidences of food insecurity.

The majority of those surveyed were Eritreans, and they represent a major group of undocumented persons and asylum seekers. It was decided not to turn only to those who had turned to Mesila (the NGO in Tel Aviv offering some assistance) as their being on Mesila’s books meant they had already sought some form of assistance and so may represent those in worse situations, and at greater risk for food insecurity. It was not possible to survey a random sample as there is no central database with contact details.

Another limitation of our study is that the data were collected in November 2020. Thus, they document the situation during the pandemic, but not following it. Nevertheless, we have reason to believe, including as reported by Mesila, that food insecurity remains a significant issue for asylum seekers and the undocumented post-pandemic, due to uncertain employment opportunities, and continuing low socioeconomic status. Accordingly, our recommendations are for developing programs that are likely to continue indefinitely.

## Summary and recommendations

The populations related to in the study do not receive the same rights as do the country's other citizens. Scant attention has been paid to their nutritional needs. Any crisis such as an economic crisis, epidemic, or war puts this population at increased risk. Given their uncertain continued employment prospects, and the ever-present danger of cessation of work, food insecurity continues to be prevalent in this population. Nutritional solutions that are not adapted to the population are not a solution. Therefore, in this study there is an attempt to adapt the nutritional solutions to the health, cultural and economic needs of the undocumented persons (workers) and asylum seekers population while maintaining their dignity, culture and the family framework in which they live. Therefore, this research proposes solutions for times of crisis, the corona pandemic and its fallout as an example of this. In addition, the recommendations and proposals are also appropriate for long term alleviation of food insecurity. The recommendations are also suitable for non-crisis times, as their economic and social circumstances create a situation where food security is not certain.

As a result of the study, we have formulated a number of recommendations for policy makers to preserve and enhance the food security of the asylum seekers and undocumented adults and to not exacerbate the health disparities. We have based some of the following recommendations on prior knowledge and contacts with these two population groups. Our recommendations include short-term and long-term interventions.

- 1. The National Food Security Council should convene a think tank, or forum, to include a nutritionist, community members, and Mesila representatives, to ensure food security and determine necessary budget sources for those with unrecognized status. The forum, convened by the National Food Security Council should include the Welfare Ministry, the Ministry of Justice, the Ministry of Education, the Ministry of Health and local municipalities, in particular those, such as Tel Aviv, with high percentages of asylum seekers. This forum will be responsible for managing government funding, and will take upon itself to monitor, in real time, implementation of the various recommendations, as listed below.
- 2. The forum should consider establishing a social-community grocery, which would supply cost-free, fresh and dried foods that are culturally suitable, to families in need, who have met predefined inclusion criteria. It could employ community members, and offer nutrition education activities, and resources, as well as specialized parental guidance for nutrition for children.
  The local authority, along with a local NGO, should take responsibility for establishment of this social-community grocery in consultation with representatives of the target population (asylum seekers), social workers, and the local District Health Office. The establishment of this facility will need adequate funding, and this should be allocated as part of the municipal budget. If this is not possible, then funding may need to be sought from the Welfare Ministry.
- 3. The newly-formed national forum (as outlined in 1) should foster cooperation with food rescue organizations, including for provision of fruit and vegetables at local food pantries and other frequented locations. There are organizations such as Leket (a national food rescue NGO) and smaller local food rescue initiatives which are very active in the field of food rescue, and contact can be made with them, to ensure provisions to these groups also.
- 4. Ministry of Health initiatives such as EfshariBari (Healthy is Possible) and local NGO's, such as Mesila in Tel Aviv, should provide guidance and training, culturally adapted, to community groups and families, including topics such as purchasing a healthy food basket on a limited budget, and how to deal with requests from children for different food types. This requires involvement of the Ministry of Education, in cooperation with the District Health Office personnel- with the Ministry as the initiator and coordinator of this effort. Consultation with the Nutrition Division of the Ministry of Health is also required for input about tailored dietary recommendations for different groups of the asylum seekers and undocumented populations. It will need to be checked whether the Welfare Ministry has counselors specializing in this field.
- 5. The Ministry of Education should map the educational institutions available to the foreign workers in the Tel Aviv area and enact a policy of supplying breakfast and hot meals to all children, where needed. The list of educational institutions needs to be maintained in real time. Although a national program is currently in place for provision of hot meals for school children, the Ministry of Education should ensure that the children of asylum seekers are included.
- 6. Distribute vouchers for use at supermarkets, with a focus on local shops. The Ministry of Justice had given some financial support during the pandemic, and it is suggested that this could continue, along with support from other ministries, such as the Welfare Ministry.

We have presented the survey results to key stakeholders, namely Mesila, the relevant persons within the Ministry of Health, and other interested parties. As a result, a limited budget from government sources, namely from the Efshari budget, has been received, to be used for the establishment of the local, community-based grocery. In addition, the Tel Aviv Municipality has participated in funding to support the work of a nutritionist to assist Mesila’s work, and a professional is now employed by the Tel Aviv Municipality, part of whose job description is to assist with food insecurity of asylum seekers and the undocumented.

## Conclusion

In Israel, during the COVID-19 pandemic, many families, including asylum seekers, were added to the pool of those suffering from food insecurity. Our study shows that asylum seekers suffered from food insecurity in large numbers during the COVID-19 pandemic. The need persists even post-pandemic. The social grocery continues—an indication that it is needed and the other recommendations are also relevant. There is a need to provide culturally adapted nutrition support and assistance. Government ministries, along with other stake holders such as food rescue organizations, local NGO’s, and local governments need to make additional efforts to include these under-franchised populations. Funds need to be budgeted for and earmarked specifically to these groups, based on well- researched and documented needs, and in consultation with community leaders. Though this study focused on an emergency situation, the recommendations listed are both for short term, emergency situations, and for long term needs, so as to ensure the health and wellbeing of these population groups.

### Supplementary Information

Supplementary Material 1.

## Data Availability

The datasets used and/or analyzed during the current study are available from the corresponding author on reasonable request.

## Footnotes

1 HCJ 4702/94, Kadam El Taiy et al. v. Minister of the Interior et al., 11 September 1995, https://www.nevo.co.il/psika_html/elyon/PADI-NH-3-843-L.htm;”Israel Report”, *The OECD Expert Group on Migration,* 2011–2012, https://m.knesset.gov.il/EN/activity/mmm/me03131.pdf.

2 In 2020 Mesila distributed 12,305 supermarket voucher cards, and 4,257 food packages.
