# Supplementary material for: Food insecurity amongst asylum seekers and people without status in Israel
Source: Isr J Health Policy Res. 2024 Aug 12;13:37. doi: 10.1186/s13584-024-00622-y (PMC11318255; doi:10.1186/s13584-024-00622-y)
Supplement: Supplementary file 1 — Supplementary Material 1. [file 13584_2024_622_MOESM1_ESM.docx]

**Food security questionnaire - refugees and migrant workers community**

The corona plague has caused many to find themselves without a job and a livelihood. During this period, many are facing nutritional challenges. This questionnaire is part of a study, in which we seek to assess the number of people in the community of refugees and migrant workers who need assistance and what type of assistance is required. Filling out the questionnaire will help us explore ways to help the community in general.

At the end of the questionnaire, we will also offer you to fill in personal details in case it is possible to get assistance from the local authority.

The researchers will not have access to the identifying data and the findings will be published without identifying details.

Thank you for filling out the short questionnaire.

1. Country of Origin: Eritrea, Sudan, Philippines, India, Former USSR, Other __________
2. Age: _______
3. Gender: male, female
4. Residential neighborhood: Ha'tikva, Shapira, Neve Shaanan, other: __________
5. No. of years in Israel: _____
6. Marital status: single, married, divorced, widowed, in an informal relationship, separated as a result of the circumstances (spouse in the country of origin)
7. How many children do you have in Israel? ____
8. Manner of residence: living alone, living with spouse or other family members, living with friends or other tenants
9. Do you get: Vouchers for food purchasing / food basket / other _________________
10. What would you rather get? Vouchers for food purchasing / dry food basket / dry food basket + fruits / vegetables / dairy and meat products
11. Do you have a refrigerator at home and means to cook and heat food? yes, no
12. Are you getting any help from an aid organization? No/ yes: which? ___________________

_

_____________________________________________________________________________

These next questions are about the food eaten in your household **in the last 6 months** and whether you were able to afford the food you need.

1 . I'm going to read you two statements that people have made about their food situation. Please tell me whether the statement was OFTEN, SOMETIMES, or NEVER true for (you/you or the other members of your household) in the last 3 months.

The first statement is, "The food that (I/we) bought just didn't last, and (I/we) didn't have money to get more." Was that often, sometimes, or never true for (you/your household) in the last 3 months?

(1) Often true

(2) Sometimes true

(3) Never true

(D, R)

______________________________________________________________________________

2 . "(I/we) couldn't afford to eat balanced meals." Was that often, sometimes, or never true for (you/your household) in the last 3 months?

(1) Often true

(2) Sometimes true

(3) Never true

(D, R)

_____________________________________________________________________________

3 . In the last 3 months, did (you/you or other adults in your household) ever cut the size of your meals or skip meals because there wasn't enough money for food?

(1) Yes

(2) No (GO TO 5)

(D, R) (GO TO 5)

______________________________________________________________________________

4. **[Ask only if # 3 = YES]** How often did this happen‑‑‑almost every month, some months but not every month, or in only 1 or 2 months?

(1) Almost every month

(2) Some months but not every month

(3) Only 1 or 2 months

(D, R)

(X) Question not asked because of negative or missing response to question 3

______________________________________________________________________________

5. In the last 3 months, did you ever eat less than you felt you should because there wasn't enough money to buy food?

(1) Yes

(2) No

(D, R)

6. In the last 3 months, were you ever hungry but didn't eat because you couldn't afford enough food?

(1) Yes

(2) No

(D, R)

Identifying information

If we can help, we will need identifying details.

The identifying data will not be exposed to the researches, but will be passed on to a representative of the

local authority for the purpose of examining the possibility of providing appropriate assistance.

**You do not have to fill out this section.**

Are you interested?

1. Yes

2. No.

If yes - enter the attached link and select a 4-digit number - first 2 digits phone number, last 2 digits date of birth

(Separate link)

Write the 4-digit number you chose - first 2 digits phone number, last 2 digits date of birth

1. Name: _________________

2. Address: ________________

3. Phone number: _________________

4. Name of the spouse (if any): ______________ Phone no.: ____________

Thank you very much for your reply. In order to reach more people from the community, we ask that you send the link to people you know from other households.
